# Assessment of Degree of Conversion and Volumetric Shrinkage of Novel Self-Adhesive Cement

**DOI:** 10.3390/polym16050581

**Published:** 2024-02-21

**Authors:** Long Ling, Yulin Chen, Raj Malyala

**Affiliations:** Glidewell Dental, Irvine, CA 92612, USA; yulinchenuc@gmail.com (Y.C.); raj.malyala@glidewelldental.com (R.M.)

**Keywords:** self-adhesive resin cements, degree of conversion, shrinkage

## Abstract

The degree of monomer conversion and polymerization shrinkage are two of the main reasons for potential adhesion failure between the tooth structure and the restoration substrate. To evaluate the degree of conversion and polymerization shrinkage of a newly developed self-adhesive resin cement, the degree of conversion (DC) was measured using FTIR under different activation modes, temperatures, and times. Volumetric shrinkage was tested using the AcuVol video imaging method. The experimental cement showed a higher DC than other cements under self-curing. The DC of the experimental cement was higher than that of other cements, except SpeedCem Plus under light curing. The experimental cement had a higher DC than other cements, except SpeedCem Plus in some conditions under dual curing. All self-adhesive cements had a higher DC at 37 °C than at 23 °C under self-curing, and there was no statistical difference between 23 °C and 37 °C under light curing. All self-adhesive cements showed a significantly higher DC at 10 min than at 5 min under self-curing. There was no statistical difference between 5 min and 10 min for most cements under dual curing. All self-adhesive cements statistically had the same volumetric shrinkage under light curing and self-curing. The newly developed self-adhesive resin cement exhibited a higher degree of conversion and similar volumetric shrinkage compared to these commercial self-adhesive resin cements.

## 1. Introduction

Self-adhesive resin cement (SARC) has gained great attention, and it has been increasingly applied since RelyX Unicem (3M ESPE) first launched in the market [1]. Compared to conventional resin cements (total-etch/esthetic and self-etch/adhesive resin cements), self-adhesive resin cement has simplified the cementation technique and process by introducing acidic monomers without separate etching or priming, resulting in lower technique sensitivity and postoperative sensitivity [1,2,3]. Like conventional resin cements, however, some factors may cause poor adhesion between the tooth structure and the restoration substrate, resulting in inferior clinical performance [4,5].

Two of the main reasons for such potential adhesion failure are the degree of monomer conversion (DC) and polymerization shrinkage [4,6,7,8,9,10]. When the monomers are polymerized, the degree of conversion represents what percentage of monomers are converted into polymers, indicating the polymerization ability or curing efficiency of resin monomers. The degree of conversion has a great influence on physical properties, like water sorption and solubility [11], mechanical properties, such as hardness, fracture toughness, and wear resistance [12,13,14], and adhesive properties [6,7,10], as well as biocompatibility [15,16]. Polymerization shrinkage happens with the decreased free volume due to the change of intermolecular forces like van der Waals into covalent bond when monomer molecules were converted to polymers. Shrinkage is widely recognized as an inherent phenomenon of polymerizable monomers, which is the clinician’s primary concern when placing resin composite restorations [17,18]. When polymerization occurs, polymerization shrinkage produces the contraction stress, and it can affect the marginal integrity and form defects at the bonding interface, resulting in marginal leakage and eventually bonding failure [17,18,19,20]. Therefore, self-adhesive resin cement with a higher degree of conversion and lower polymerization shrinkage is always highly desirable.

Recently, we reported a novel self-adhesive resin cement (SARC) with favorable physical, mechanical, and adhesive properties and evaluated its working time and setting time, consistency, film thickness, water sorption and solubility, flexural strength and modulus, and bond strength [21]. This study aims to investigate the degree of conversion and volumetric shrinkage of the newly developed self-adhesive resin cement and compare it with other commercial self-adhesive resin cements. The hypothesis is that this new self-adhesive resin cement has a higher degree of conversion, especially for self-curing, and similar volumetric shrinkage compared to other commercial self-adhesive resin cements.

## 2. Materials and Methods

### 2.1. Study Design

In this in vitro study, a newly developed self-adhesive resin cement, as an experimental cement, and five commercially available self-adhesive resin cements, as control materials, were examined to compare the degree of conversion (DC) and volumetric shrinkage. The degree of conversion was studied in detail at different activation modes (self-cure (SC), light cure (LC), and dual cure (DC)), temperatures (23 °C and 37 °C), and times (5 min and 10 min for self-cure, 20 s for light cure) based on a comprehensive consideration of ISO 4049 [22], a clinic cementation protocol, and the work and set times of most resin cements [21,23,24,25,26,27]. Therefore, 12 curing scenarios were set up, as shown in Table 1. Volumetric shrinkage was collected for self-cure at 60 min and for light cure at 5 min. All testing materials and methods are described in detail below.

### 2.2. Materials

Experimental self-adhesive resin cement (Experimental SARC or Experimental) was formulated using our proprietary adhesive resin and filler technology, which included acidic monomer, non-acidic monomers, dual-cured initiator systems, inhibitor, and fillers. 10-methacryloxydecyl dihydrogen phosphate (10-MDP) as an acidic monomer, bisphenol A diglycidyl ether dimethacrylate (BisGMA) as rigid space-filling monomers, and other dental monomers, such as urethane dimethacrylate, were used for the resin system. Dual-curing initiator systems were composed of cumene hydroperoxide/(2,3-difluorophenyl)thiourea as a redox initiator system for self-curing and camphorquinone (CQ), bis(2,4,6-trimethylbenzoyl)-phenyl-phosphineoxide, and ethyl 4-dimethylaminobenzoate as photo-initiators for light curing. 2,6-di-(tert-butyl)-4-methylphenol was used as an inhibitor. Fillers consisted of barium boron fluoroaluminosilicate glass, OX-50 fumed silica, and ytterbium fluoride. The homogeneous resin mixtures were first obtained by stirring resin monomers with the additives until dissolved. The resulting resin mixtures were further mixed with fillers until a uniform, flowable paste was formed. Five commercially available self-adhesive resin cements, including Maxcem Elite (Kerr, Orange, CA, USA), RelyX Unicem 2 (3M ESPE, St. Paul, MN, USA), SpeedCem Plus (Ivoclar Vivadent, Schaan, Liechtenstein), SmartCEM 2 (Dentsply Sirona, York, PA, USA), and Calibra Universal (Dentsply Sirona, York, PA, USA), major self-adhesive resin cements with some similar resins, fillers, and filler loading in the market, were selected in this study for comparison. Further information about the self-adhesive resin cements used in this study is shown in Table 2.

### 2.3. Degree of Conversion (DC)

The degree of conversion was measured using a Bruker ALPHA FTIR spectrometer with a Diamond Crystal ATR (Bruker, Billerica, MA, USA). Disk specimens (10 mm diameter × 1 mm thick, n = 5) were placed directly on the diamond crystal plate and cured in situ at different activation modes (self-cure, light cure, and dual cure), temperatures (23 °C and 37 °C), and times (5 min and 10 min for self-cure, 20 s for light cure). All spectra were recorded for uncured and cured cements at a wavelength range of 4000–400 cm^−1^, a resolution of 4 cm^−1^, and a sample scan time of 24 s. The DC was calculated with the peak heights of aliphatic double bond absorption around 1638 cm^−1^ and of the aromatic double bond around 1607 cm^−1^ as the internal standard (for most cements; otherwise, the carbonyl group around 1710 cm^−1^ for cement without absorption around 1607 cm^−1^ was used).

DC (%) = [1 − (A_p_/A_p0_)/(A_m_/A_m0_)] × 100

where A_p_ is the peak height of the cured cement (polymer) and A_m_ is the peak height of uncured cement (monomer) at 1638 cm^−1^ and A_m0_ and A_p0_ are the peak heights at 1607 cm^−1^ (or 1710 cm^−1^) before and after curing, respectively.

### 2.4. Volumetric Shrinkage

Volumetric shrinkage (%) was measured using an AcuVol 2 device (Bisco, Inc. Schaumburg, IL, USA). Bubble-free, semispherical specimens of resin materials were manually formed by carefully placing the specimen (approximately 20 mg) onto the base of the sample holder. The curing gun was placed approximately 2 mm from the specimen and light cured for 20 s with a light intensity of approximately 1000 mW/cm^2^ (Bluephase Style, Ivoclar Vivadent AG, Schaan, Liechtenstein). The volumetric shrinkage of each resin material (n = 5) was obtained at 5 min for light curing and at 60 min for self-curing to ensure that the volumetric shrinkage became constant at 5 min for light curing and at 60 min for self-curing.

### 2.5. Flexural Strength, Flexural Modulus, Water Sorption, and Solubility

Flexural strength, flexural modulus, water sorption, and solubility were determined in accordance with ISO-4049 (2009) in our previous study [21].

### 2.6. Statistical Analysis

Statistical analysis was performed using Minitab 18 Statistical Software (Minitab, LLC, State College, PA, USA). The results for the degree of conversion and volumetric shrinkage were analyzed through one-way ANOVA and Tukey’s post hoc comparison. The significance level was set at α = 0.05. The Pearson correlations were analyzed according to the simple linear regression between the degree of conversion and flexural strength, flexural modulus, and water solubility.

## 3. Results

**Figure 1:**
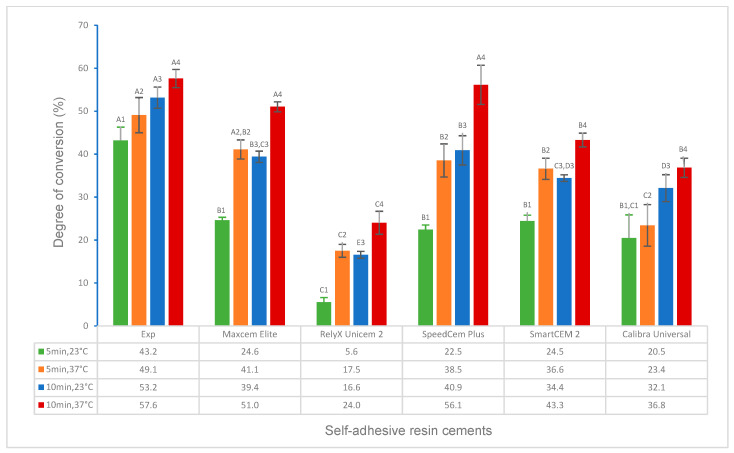
Degree of conversion of self-adhesive resin cements for self-curing (5 min and 10 min at 23 °C and 37 °C). (Value bars with the same letter are statistically equivalent between the tested groups).

**Figure 2:**
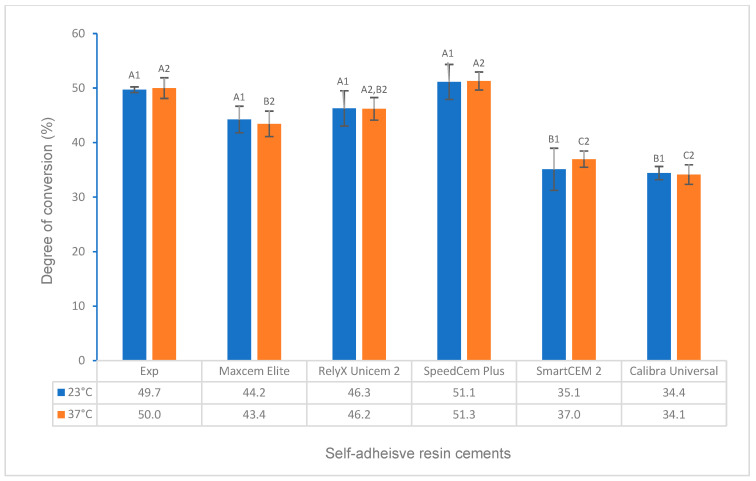
Degree of conversion of self-adhesive resin cements for light curing (20 s at 23 °C and 37 °C). (Value bars with the same letter are statistically equivalent between the tested groups).

**Figure 3:**
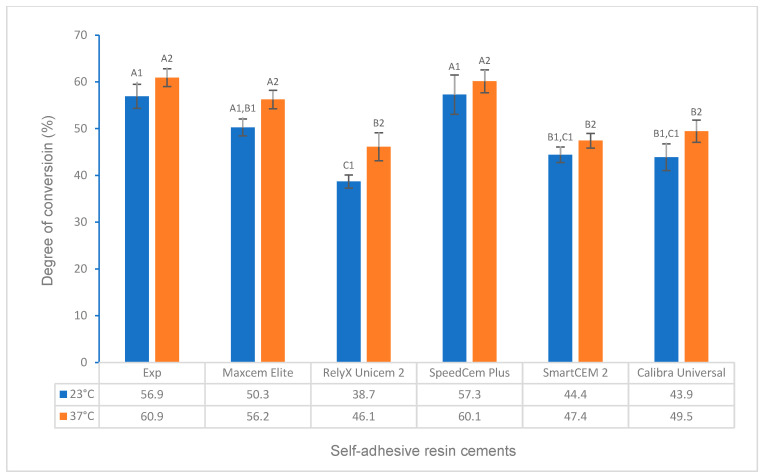
Degree of conversion of self-adhesive resin cements for dual curing (self-curing for 10 min plus light curing for 20 s at 23 °C and 37 °C). (Value bars with the same letter are statistically equivalent between the tested groups).

**Figure 4:**
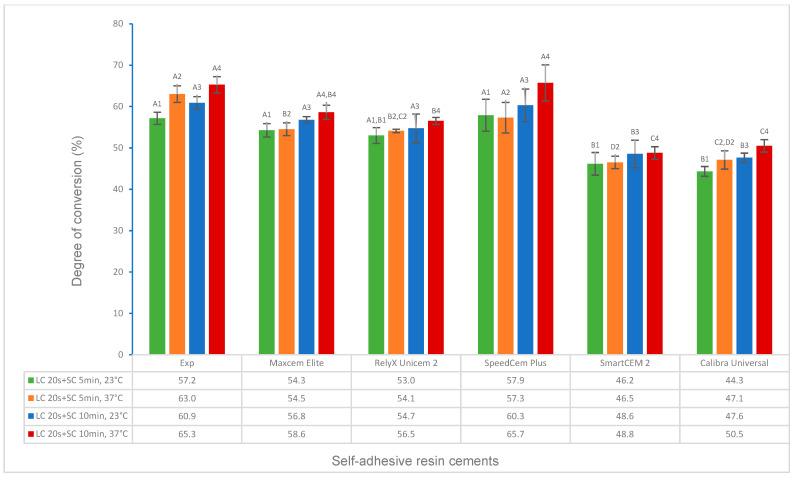
Degree of conversion of self-adhesive resin cements for dual curing (light curing for 20 s plus self-curing for 5 min and 10 min at 23 °C and 37 °C). (Value bars with the same letter are statistically equivalent between the tested groups).

**Figure 5:**
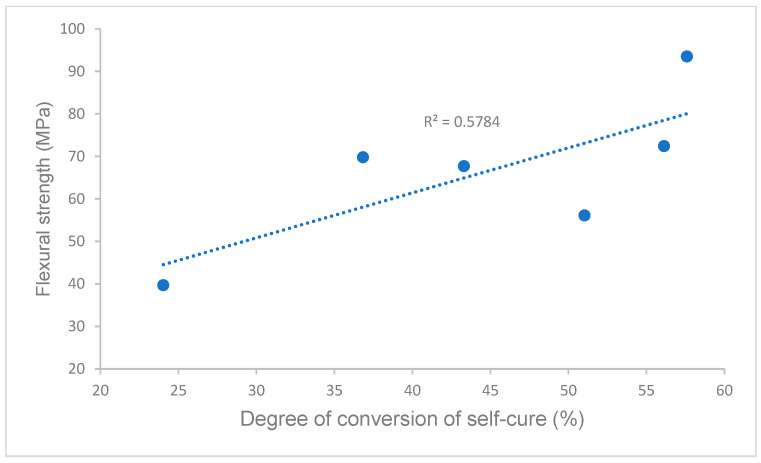
Correlation of degree of conversion and flexural strength.

**Figure 6:**
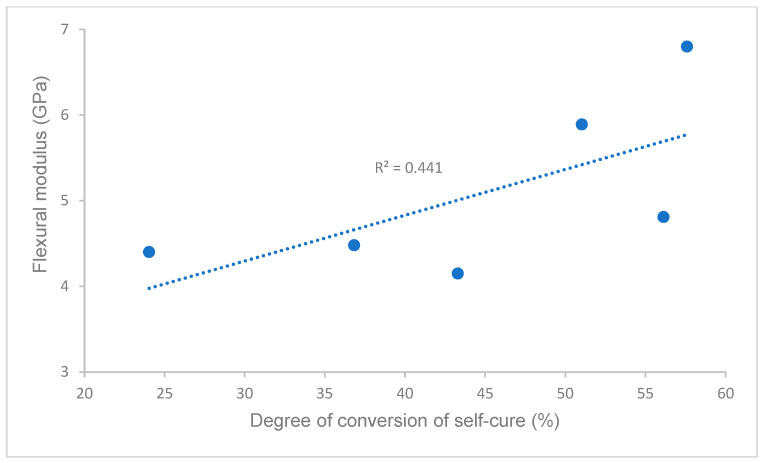
Correlation of degree of conversion and flexural modulus.

**Figure 7:**
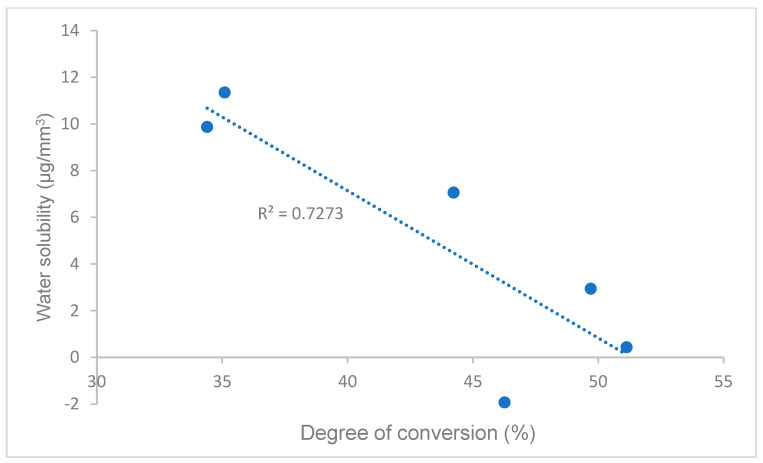
Correlation of degree of conversion and water solubility.

**Figure 8:**
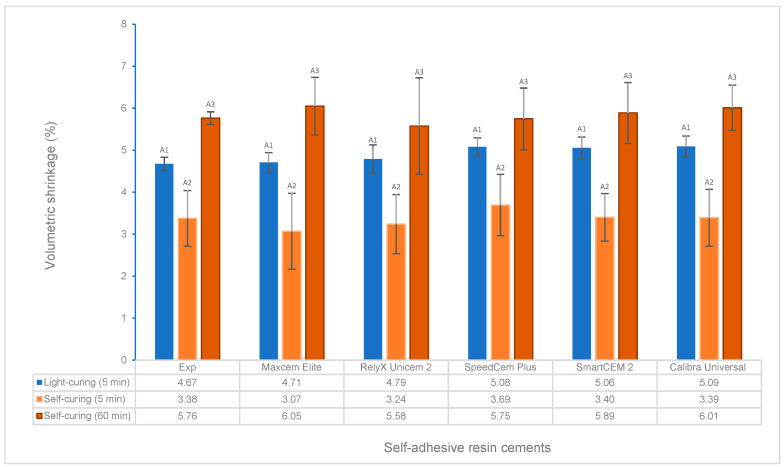
Volumetric shrinkage for light curing at 5 min and self-curing at 5 min and 60 min. (Value bars with the same letter are statistically equivalent between the tested groups).

**Figure 9:**
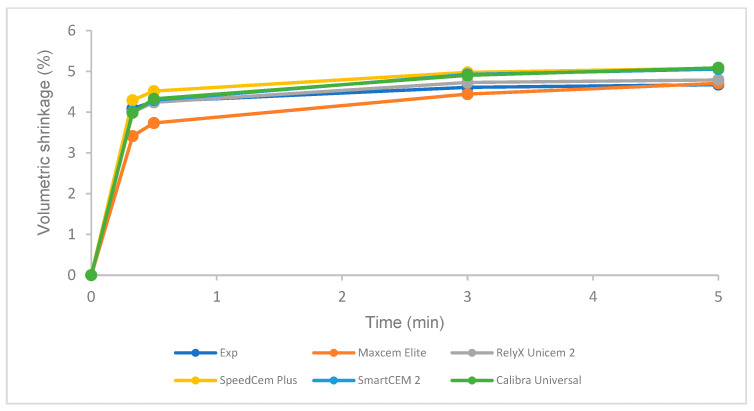
Volumetric shrinkage of self-adhesive resin cements over time for light curing.

**Figure 10:**
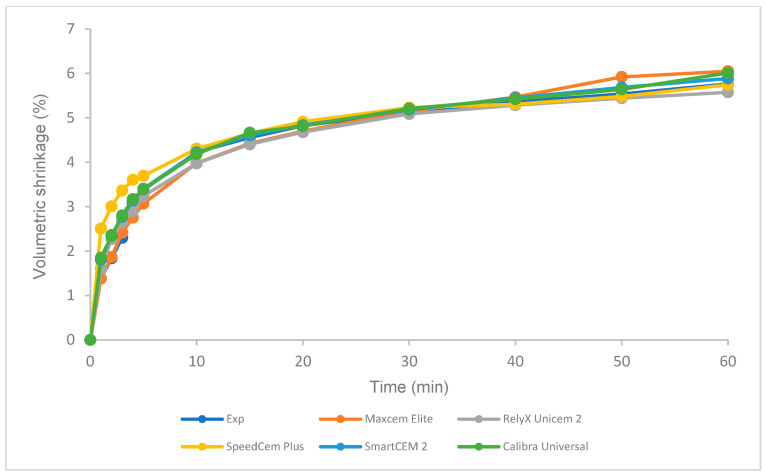
Volumetric shrinkage of self-adhesive resin cements over time for self-curing.

The test results for the degree of conversion and volumetric shrinkage of experimental and commercially available self-adhesive resin cements, which include the effects of activation modes, temperature, and time, and the correlations between DC and flexural strength, modulus, and water solubility, respectively, are shown in Figure 1, Figure 2, Figure 3, Figure 4, Figure 5, Figure 6, Figure 7, Figure 8, Figure 9 and Figure 10.

### 3.1. Effect of Cement Materials on DC under Different Activation Modes

Under self-curing for 5 min and 10 min at 23 °C and 37 °C (Table 1, curing scenarios 1–4), the Experimental SARC showed a significantly higher DC than other cements (*p* < 0.001); however, statistically, it was the same as Maxcem Elite and SpeedCem Plus for 10 min at 37 °C (*p* > 0.05) (Figure 1).

Under light curing for 20 s at 23 °C and 37 °C (Table 1, curing scenarios 5 and 6), the DC of the Experimental SARC was statistically the same as that of Maxcem Elite, RelyX Unicem 2, and SpeedCem Plus at 23 °C and SpeedCem Plus at both 23 °C and 37 °C (*p* > 0.05). It was significantly higher than SmartCEM 2 and Calibra Universal at 23 °C and Maxcem Elite, RelyX Unicem 2, SmartCEM 2, and Calibra Universal at 37 °C (*p* < 0.001) (Figure 2).

For dual curing at different temperatures, times, and orders of self-curing and light curing, the Experimental SARC had a significantly higher or higher DC than other cements (*p* < 0.001), except SpeedCem Plus at 23 °C and Maxcem Elite and SpeedCem Plus at 37 °C (*p* > 0.05) (Figure 3) in curing scenarios 7 and 8 (Table 1, SC 10 min + LC 20 s at 23 °C and 37 °C). Compared to curing scenarios 7 and 8, the Experimental SARC showed a significantly higher or higher DC than RelyX Unicem 2, SmartCEM 2, and Calibra Universal at 23 °C, and it was significantly higher than that of other cements (*p* < 0.001), except SpeedCem Plus (*p* > 0.05) at 37 °C (Figure 4) in curing scenarios 9 and 10 (LC 20 s + SC 10 min at 23 °C and 37 °C). Compared to curing modes 9 and 10, the DC of the Experimental SARC was statistically the same as that of Maxcem Elite, RelyX Unicem 2, and SpeedCem Plus (*p* > 0.05), but it was significantly higher than that of SmartCEM 2 and Calibra Universal (*p* < 0.001) at 23 °C and significantly higher or higher than that of other cements (*p* < 0.001), except SpeedCem Plus at 37 °C (*p* > 0.05) (Figure 4) in curing scenarios 11 and 12 (LC 20 s + SC 5 min at 23 °C and 37 °C).

### 3.2. Effect of Temperature on DC under Different Activation Modes

For all self-adhesive cements in self-curing (curing scenarios 1–4), the DC at 37 °C was significantly higher or higher than that at 23 °C (Figure 1). For all self-adhesive cements in light curing (curing scenarios 5 and 6), there was no statistical difference between 23 °C and 37 °C for any of the self-adhesive cements (Figure 2). For dual curing, the DC at 37 °C was significantly higher for Maxcem Elite and RelyX Unicem 2 (*p* < 0.001), and it was higher for other cements (*p* > 0.05) than that at 23 °C (Figure 3) in curing scenarios 7 and 8. There was no statistical difference between 23 °C and 37 °C for any of the cements except the Experimental SARC (Figure 4) in curing scenarios 9–12.

### 3.3. Effect of Time on DC under Different Curing Scenarios

All self-adhesive cements showed a significantly higher DC for 10 min than for 5 min at both 23 °C and 37 °C in self-curing (Figure 1). For all cements under dual curing, there was no statistical difference between 5 min and 10 min except for the Experimental SARC and Calibra Universal at 23 °C and RelyX Unicem 2 at 37 °C (*p* < 0.001) *(*Figure 4).

### 3.4. Correlations between the Degree of Conversion and Flexural Strength, Flexural Modulus, and Water Solubility

Pearson’s correlations between the degree of conversion and flexural strength, flexural modulus, and water solubility in self-curing (data of flexural strength, flexural modulus, and water solubility are from our previous study [21]) are shown in Figure 5, Figure 6 and Figure 7. A strong or moderate correlation was found between the degree of conversion and flexural strength (Pearson’s correlation coefficient R = 0.7605), flexural modulus (R = 0.6641), and water solubility (R = 0.8528) according to Evans’ and Moore’s guide (R > 0.6 or 0.7 indicated moderate or strong relationship) [28,29].

### 3.5. Volumetric Shrinkage under Different Curing Modes

There was no statistical difference in volumetric shrinkage among these tested cements under light curing at 5 min and self-curing at 5 min and 60 min, respectively (Figure 8). For each cement, the volumetric shrinkage of self-curing was significantly lower than that of light curing after 5 min at 23 °C (Figure 8). Self-curing showed a slower increase in volumetric shrinkage than light curing over time (Figure 9 and Figure 10).

## 4. Discussion

A couple of testing methods have been used to determine the degree of conversion, like Fourier transform infrared (FTIR) [30,31,32], Raman spectroscopy [33], Stray-field magnetic resonance imaging (STRAFI-MRI) [34], Photo differential scanning calorimetry (photo-DSC) [35], etc. FTIR is the most common method for measuring the DC in most studies [30,31,32], which was used to determine the DC at various curing scenarios in this study.

Self-curing (auto-polymerization/chemically activated) is essential for most indirect restorations, especially for metal or ceramo-metal inlays, onlays, crowns and bridges, and endodontic post, because little or no light is transmittable through the restorative materials. For all curing scenarios of self-curing (curing scenarios 1–4, 5 min and 10 min at 23 °C and 37 °C), the Experimental SARC showed a significantly higher or higher DC than other cements (Figure 1), while RelyX Unicem 2 had the lowest value of DC, which (16.6 ± 0.8% at 23 °C for 10 min) was similar to the values the literature reported (11.05 ± 4.16% at 23 °C for 10 min) [25]. The different values of DC are attributed to the cement composition, primarily the chemical composition of resin matrices, such as self-curing initiator systems, inhibitors, and monomers [25,35,36,37]. Table 2 lists the resin composition of all cements based on the available information, indicating some differences among these cements. The Experimental SARC was formulated through our proprietary resin and filler technology, which uses cumene hydroperoxide/(2,3-difluorophenyl)thiourea as the self-curing initiator system. RelyX Unicem 2 and SpeedCem Plus contained sodium persulfate and dibenzoyl peroxide, respectively [25], whereas no information is available for Maxcem Elite, SmartCEM 2, or Calibra Universal. In addition, the acidic monomer, as an important component of self-adhesive resin cement, influences the initiator system, especially for the initiator system containing the amine co-initiator [25]. The Experimental SARC and SpeedCem Plus used 10-methacryloxydecyl dihydrogen phosphate (10-MDP) as a self-etch and adhesive monomer in their resin composition, while Maxcem Elite, SmartCEM 2, and Calibra Universal used glycerol phosphate dimethacrylate (GPDM) and dipentaerythritol pentaacrylate monophosphate (PENTA)/4-methacryloxyethyl trimellitate anhydride (4-META), respectively. The Experimental SARC used cumene hydroperoxide/(2,3-difluorophenyl)thiourea as self-curing initiators instead of the amine system. All of these resulted in different values of DC, affecting the cement properties, such as physical and mechanical properties [11,12,13,14]. This was proven in this study by a strong correlation between the DC and the flexural strength (R = 0.7605) or flexural modulus (R = 0.6641) in self-curing (Figure 5 and Figure 6), i.e., the Experimental SARCs exhibited statistically higher flexural strength and flexural modulus than other cements, whereas RelyX Unicem 2 had the lowest flexural strength and flexural modulus among these cements [21].

Clearly, curing temperature and time have a great effect on DC for self-curing (Figure 1). All self-adhesive cements in the present study showed a significantly higher or higher DC at 37 °C than at 23 °C for both 5 min and 10 min, and the DC for 10 min was significantly higher than that at 5 min at both 23 °C and 37 °C. It is easily understood that higher temperatures and longer times will result in more monomers converted to polymers by reducing the viscosity of the reaction system and increasing the polymerization rate according to the Arrhenius equation, free radical polymerization theory, and the literature reported [4,38,39].

For light curing at both 23 °C and 37 °C (Figure 2), the DC of the Experimental SARC and SpeedCem Plus were higher than other cements, and RelyX Unicem 2 had a significant increase from self-curing to light curing. The findings are mainly related to the light curing initiator system [31,40,41]. The Experimental SARC contains both camphorquinone (CQ, Absmax at 470 nm) and bis(2,4,6-trimethylbenzoyl)-phenyl-phosphineoxide (BTPPO, Absmax at 365 nm) as photo-initiators, which can cover a wider range of light absorption from Ultraviolet (UV) to visible (blue) regions (320–500 nm) compared to only CQ used for some cements [41]. The curing light gun (Bluephase Style, Ivoclar Vivadent AG, Schaan, Liechtenstein) used in this study can cure without restriction in the wavelength range of 380 to 515 nm. The optimal combination resulted in a higher DC. The other cements, like SpeedCem Plus, may use other high-efficiency photo-initiator systems, resulting in a higher DC as well. The results of our previous water solubility test [21] strongly support the different performances of DC among these cements. DC has a strong negative relationship with water solubility in this study [Figure 7], i.e., the higher the DC, the less monomers existed, which formed more densely cross-linked networks and thus resulted in the potentially lower water solubility.

For all self-adhesive cements under light curing (curing scenarios 5 and 6), there was no statistical difference between 23 °C and 37 °C (Figure 2), indicating that temperature did not affect the DC between room temperature (23 °C) and oral temperature (37 °C).

Compared to self-curing, all cements had a significantly higher DC in light curing for 20 s at 23 °C and 37 °C (curing scenarios 5 and 6) than in self-curing for 5 min at 23 °C (curing scenario 1), whereas each cement behaved differently compared to other self-curing scenarios (curing scenario 2–4, 10 min at 23 °C and 5 min and 10 min at 37 °C). For example, only RelyX Unicem 2 showed a significantly higher DC in light curing than in all scenarios of self-curing, which means that RelyX Unicem 2 depends mainly on photoactivation and needs a longer time at oral temperature if self-cured. The DCs of the Experimental SARC for light curing at 23 °C and 37 °C were lower than the self-curing DC for 10 min at 23 °C and 37 °C, respectively, which was similar to 5 min at 37 °C and higher than 5 min at 23 °C.

Resin cements were developed in dual-cure activation mode to ensure higher curing efficiency (a higher DC) for various restorations by combining the benefits of both light curing and self-curing to obtain favorable physical, mechanical, and bonding properties [25]. For all cements, each cement had a significantly higher or higher dual-curing DC (curing scenarios 7–12) than its corresponding self-curing DC (curing scenarios 1–4), except that statistically, the Experimental cement and the Maxcem Elite had the same DC for self-curing for 10 min at 37 °C (scenario 4) as first self-curing for 10 min and then light curing for 20 s at 23 °C (scenario 7) (Experimental and Maxcem Elite) and first light curing for 20 s and then 5 min of self-curing (scenario 11) (Experimental). The results of this study are in agreement with other studies reported, which indicated that dual-curing mode is more effective than self-curing mode, i.e., generally, dual curing has a much higher DC than self-curing [25,42]. The Experimental cement exhibited a significantly higher or higher DC than other cements except, statistically, the same value as SpeedCem Plus in dual-curing mode, but the difference in the DC between the Experimental cement and other cements decreased compared to self-curing mode due to the stronger energetic curing promoted by light curing. In addition, the order of self-curing and light curing (immediate or delayed light curing) in the dual-curing mode also affects the DC. The DC of immediate light curing (first 20 s of light curing and then 10 min of self-curing, curing scenarios 9 and 10) in this study was slightly higher or higher than that of delayed light curing (first 10 min of self-curing and then 20 s of light curing, curing scenarios 7 and 8) when cements started to mix, depending on the materials (composition), temperature, time, etc. [34,43]. This is because light curing has a much higher polymerization rate than self-curing, and it can quickly reach a high monomer conversion (DC) due to the different initiator systems when cements are immediately exposed to light once mixed [43].

Shrinkage is widely recognized as an inherent phenomenon in polymerizable monomer resins when polymerization occurs [18,19,20]. There have been different methods to measure the volumetric shrinkage of composite resins, such as the use of the mercury dilatometer, the Archimedes method, and the video imaging method [44,45,46,47,48]. AcuVol is a video imaging measurement system that allows for noncontact visual analysis of volume changes in small-volume samples, and it determines volumetric shrinkage in real time based on optical measurements and pattern recognition [47,48]. Analysis can be conducted in actual volume units and as percentage changes in volume. Volumetric shrinkage in this study was evaluated using AcuVol.

The volumetric shrinkage of the Experimental cement was statistically the same as that of other cements under light curing at 5 min and self-curing at 5 min and 60 min, respectively (Figure 8). The volumetric shrinkage mainly depends on the composition of the resin cements under the same curing conditions, i.e., the resin matrix, such as monomer type/structure and initiators, and the filler, such as loading, type, and size [49,50,51,52]. Based on the limited information provided by the manufacturers regarding the composition of the tested self-adhesive cements (Table 2), most cements have the same or similar filler loading (69–70%), yet some of the fillers and resins are the same, such as barium boron fluoroaluminosilicate, amorphous silicon dioxide, and urethane dimethacrylate (UDMA); this is probably the major reason that these cements showed similar volumetric shrinkage under light curing and self-curing.

Like resin-based composites, manufacturers have tried to develop low-shrinkage, self-adhesive cements to reduce the risk of marginal leakage and bonding failure while achieving a proper or higher DC. A higher DC often follows higher shrinkage, which causes increased shrinkage stress that could exceed the bond strength between the tooth structure and the restoration substrate, resulting in potential de-bonding/cementation failure [11,53]. As an increasing DC leads to increased shrinkage, an optimal DC and minimal shrinkage are expected and can be possibly achieved by optimizing the composition of resin materials (monomer structure and filler, curing technology, etc.) [53,54]. The Experimental SARC was formulated using our proprietary resin and filler technology, which is based on low-shrinkage monomers, such as ethoxylated bisphenol A dimethacrylate with higher molecular weight and lower viscosity, with consideration of physical, mechanical, and adhesive properties. A higher DC was obtained with similar volumetric shrinkage compared to other cements. Clearly, the volumetric shrinkage of self-curing is significantly lower than that of light curing for all cements at 5 min (Figure 8), and self-curing has a lower polymerization rate than light curing (Figure 8 and Figure 9), which is in agreement with previous studies due to the difference in the initiator system and the curing mode/mechanism of self-curing and light curing [38,52,55].

The limitation is that this study did not consider the polymerization kinetics of monomer conversion, like the rate of polymerization, and polymerization shrinkage stress. Future work will focus on these investigations.

## 5. Conclusions

The hypothesis has been proven, i.e., a newly developed, self-adhesive resin cement exhibited a higher degree of conversion and similar volumetric shrinkage compared to the commercial self-adhesive resin cements based on the findings of this study for the degree of conversion and volumetric shrinkage. Experimental self-adhesive resin cement is a good alternative to a simplified clinic bonding procedure for the cementation of indirect restoration.

## Figures and Tables

**Table 1:** Curing scenarios of self-adhesive resin cements used in this study.

Curing Scenarios	Self-Cure (SC)	Light Cure (LC)	Dual Cure (DC)
1	5 min at 23 °C		
2	5 min at 37 °C		
3	10 min at 23 °C		
4	10 min at 37 °C		
5		20 s at 23 °C	
6		20 s at 37 °C	
7			SC 10 min + LC 20 s at 23 °C
8			SC 10 min + LC 20 s at 37 °C
9			LC 20 s + SC 10 min at 23 °C
10			LC 20 s + SC 10 min at 37 °C
11			LC 20 s + SC 5 min at 23 °C
12			LC 20 s + SC 5 min at 37 °C

**Table 2:** Self-adhesive resin cements used in this study.

Material	Manufacturer	Resin	Filler	Filler Content (wt.%)
Exp. SARC	Glidewell	BisGMA, UDMA, TEGDMA, MDP, initiators and inhibitor	Barium boron fluoroaluminosilicate glass, fumed silica, ytterbium fluoride	70
MaxCem Elite	Kerr	Methacrylate esters, GPDM, HEMA, activators and stabilizers	Mineral fillers, ytterbium fluoride	69
RelyX Unicem 2	3M ESPE	Methacrylated phosphoric esters, dimethacrylates, TEGDMA, acetate, sodium persulfate, substituted pyrimidine, stabilizers	Glass fillers, silica, calcium hydroxide	70
SpeedCEM Plus	Ivoclar Vivadent	UDMA, TEGDMA, PEGDMA, DDDMA, MDP, dibenzoyl peroxide, stabilizer	Barium glass, silica ytterbium trifluoride	75 (Base) /69.8 (Cat)
SmartCEM 2	Dentsply Sirona	UDMA, EBPADMA, Di- and tri-functional function diluents, PENTA, 4-META, initiators, accelerators, stabilizer	Barium boron fluoroaluminosilicate glass, amorphous silicon dioxide	69
Calibra Universal	Dentsply Sirona	UDMA, Di- and Tri-Methacrylate, Phosphoric acid modified acrylate, initiators, accelerators, stabilizer, BHT	Barium boron fluo roaluminosilicate, amorphous silicon dioxide	73

BisGMA—Bisphenol A diglycidyl ether dimethacrylate; UDMA—Urethane dimethacrylate; TEGDMA—Triethyleneglycol dimethacrylate; MDP—Methacryloyloxydecyl dihydrogenphosphate; GPDM—Glycerol phosphate dimethacrylate; HEMA-2—Hydroxyethyl methacrylate; PEGDMA—Polyethylene glycol dimethacrylate; DDDMA-1,10—decandiol dimethacrylate; EBPADMA—Ethoxylated Bis Phenol A Dimethacrylate; PENTA—Dipentaerythritol penta-acrylate monophosphate; 4-META-4—Methacryloxyethyl trimellitate anhydride; CQ—camphorquinone; EDMAB—ethyl 4-dimethylaminobenzoate; BHT—Butylated hydroxytoluene. The compositions of the resin and filler were obtained from the manufacturers.

## Data Availability

Data are contained in the article.
